# The Dichloromethane Fraction of *Croton sonorae*, A Plant Used in Sonoran Traditional Medicine, Affect *Entamoeba histolytica* Erythrophagocytosis and Gene Expression

**DOI:** 10.3389/fcimb.2021.693449

**Published:** 2021-07-23

**Authors:** Isaac Villegas-Gómez, Angélica Silva-Olivares, Ramón Enrique Robles-Zepeda, Juan-Carlos Gálvez-Ruiz, Mineko Shibayama, Olivia Valenzuela

**Affiliations:** ^1^ Departamento de Ciencias Químico Biológicas, Universidad de Sonora, Hermosillo, México; ^2^ Departamento de Infectómica y Patogénesis Molecular, Centro de Investigación y de Estudios Avanzados del Instituto Politécnico Nacional (CINVESTAV-IPN), Ciudad de México, México

**Keywords:** *Entamoeba histolytica*, *Croton sonorae*, amoebicidal effect, erythrophagocytosis, ultrastructural changes, gene expression

## Abstract

Intestinal parasites are a global problem, mainly in developing countries. Obtaining information about plants and compounds that can combat gastrointestinal disorders and gastrointestinal symptoms is a fundamental first step in designing new treatment strategies. In this study, we analyzed the antiamoebic activity of the aerial part of *Croton sonorae*. The dichloromethane fraction of *C. sonorae* (CsDCMfx) contained flavonoids, terpenes, alkaloids, and glycosides. The ultrastructural morphology of the amoebae treated for 72 h with CsDCMfx was completely abnormal. CsDCMfx reduced erythrophagocytosis of trophozoites and the expression of genes involved in erythrocyte adhesion (*gal/galnac lectin*) and actin cytoskeleton rearrangement in the phagocytosis pathway (*rho1 gtpase* and *formin1*). Interestingly, CsDCMfx decreased the expression of genes involved in *Entamoeba histolytica* trophozoite pathogenesis, such as cysteine proteases (*cp1*, *cp4*, and *cp5*), *sod*, *pfor*, and *enolase.* These results showed that *C. sonorae* is a potential source of antiamoebic compounds.

## Introduction

Parasitic intestinal infections have medical and economic impacts worldwide, and it is estimated that three billion people are affected annually. *Entamoeba histolytica* is one of the most prevalent intestinal protozoa in the human gut and is the etiologic cause of amoebiasis and amoebic liver abscess (ALA), affecting approximately 50 million people and approximately 100,000 deaths annually (Stanley, 2003). Amoebiasis is endemic in Mexico, and 187,785 cases were registered in 2019 (Secretaría De Salud, 2019). *E. histolytica* infection is established by parasite adherence to the colonic mucin layer and is capable of invasion of the large intestine, causing extensive tissue destruction and an important inflammatory reaction (Marie and Petri, 2014). Without treatment, it can result in amoebic dysentery and ALA (Cornick et al., 2017). Metronidazole (MTZ) is the most commonly used drug for *E. histolytica* intestinal infection and liver necrosis. In addition, strains resistant to MTZ have been reported, and MTZ can cause different secondary effects, such as diarrhea, nausea, headache, and teratogenic effects (Penuliar et al., 2015).

Through many generations, plants have been used to treat different diseases and their symptoms, including gastrointestinal disorders such as diarrhea, nausea, stomachache, colitis, and vomiting, which can be caused by parasites such as *E. histolytica * (Mi-Ichi et al., 2016). In Sonoran traditional medicine, some ethnic groups, such as Mayos, Yaquis, Seris, Pimas, and Guarijios, have used certain plants to treat gastrointestinal disorders and gastrointestinal symptoms (Moreno-Salazar et al., 2008). Some compounds isolated from the *Croton* species, mostly terpenoids, glycosides, alkaloids, a few flavonoids and others (dos Santos et al., 2015; Xu et al., 2018), have antiproliferative, antifungal, antibacterial and antiparasitic activities (Noor Rain et al., 2007; Adelekan et al., 2008; Guimarães et al., 2010; Obey et al., 2018). In the *Croton* genus, more than 300 terpenoids have been identified and characterized (Xu et al., 2018). Velázquez-Domínguez et al. (2013) reported a sesquiterpene lactone incomptine A, with an amoebicidal effect on *E. histolytica.* Other reports have demonstrated *in vitro* amoebicidal properties of essential oils of the aerial parts of some *Croton* species (rich on sesquiterpenes) against trophozoites of *Acanthamoeba polyphaga* (Vunda et al., 2012). Flavonoids (Resveratrol) can induce oxidative stress, dysregulation of glycolytic enzymes, and apoptosis-like death in *E. histolytica* trophozoites (Pais-Morales et al., 2016). Additionally, some studies have shown that the main molecular targets correspond to cytoskeleton-related proteins such as myosin II, actin and α-actin, modifying pathogenic mechanisms such as adhesion, cytolysis, phagocytosis and migration (Bolaños et al., 2014; Bolaños et al., 2015; Pais-Morales et al., 2016).

The actin cytoskeleton is an important virulence factor in *E. histolytica* that is involved in phagocytosis for nutrient intake and invasion (Aslam et al., 2012). Rho GTPases and their downstream effectors, such as formin, regulate cytoskeletal reorganization in various cellular processes, such as cytokinesis, motility, and apoptosis (Hall, 1998). *E. histolytica* expresses a family of eight *formins*; Ehformin 1 and 2 have been shown to interact with microtubule assembly in the nucleus and regulate mitosis and cytokinesis in *E. histolytica* (Majumder and Lohia, 2008). Approximately 50 cysteine proteases (*cps*) genes present in the *E. histolytica* genome (Irmer et al., 2009), and the expression is different when the trophozoites analyzed were obtained from *in vitro *culture versus *in vivo* infection (He et al., 2010) and are essential for mucus degradation and for the degradation of phagocytosed cells or debris (Nakada-Tsukui et al., 2012). The *E. histolytica* death process is not well understood, and this organism has non-canonical caspases (Pais-Morales et al., 2016), but calpain-like protein activity increases when programmed cell death (PCD) is induced by nitric oxide species (NOS) (Ramos et al., 2007; Villalba et al., 2007; Nandi et al., 2010; Domínguez-Fernández et al., 2018). In this study, we analyzed the antiamoebic activity of the aerial part of *Croton sonorae*. The dichloromethane fraction of *C. sonorae* contained the most effective compounds against *E. histolytica* trophozoites, demonstrating an effect on erythrophagocytosis and affecting the gene expression of some proteins involved in the pathogenesis of this amoebae.

## Materials and Methods

### Plant Material

*Croton sonorae* was collected at the location 29°09’03.00” N, 110°56’59.51” W, with classification number 21,420. This plant was authenticated by Professor José Jesús Sánchez Escalante, and a voucher specimen number was later deposited in the Herbarium of the University of Sonora.

### Extraction of *C. sonorae*

The aerial part of *C. sonorae* was dried at room temperature for at least 2 weeks. The dried plant material was powdered and mixed with methanol (1:10 w/v) for 10 days, and the resulting extract was evaporated under reduced pressure (Jiménez-Estrada et al., 2013). The *C. sonorae* extract was fractionated using methanol, hexane, ethyl acetate and dichloromethane (DCM).

### Amoebic Culture

*E. histolytica* HM1:IMSS, kindly donated by Dr. Mineko Shibayama, was cultured axenically at 37°C in TYI-S-33 medium supplemented with 20% (v/v) heat-inactivated bovine serum and 10% (v/v) diamond vitamin-Tween 80 solution (Diamond et al., 1978).

### Growth Curve of *E. histolytica* Trophozoites

Trophozoites in the log phase of growth were placed on 24-well plates with different initial inoculum, 4 × 10^4^, 6 × 10^4^, 8 × 10^4^ and 1 × 10^5^ trophozoites/well in triplicate (were kept in BD GasPack jars), and counted in a hemocytometer every 24 until 96 h. Viability was measured with trypan blue exclusion dye (Penuliar et al., 2015).

### Antiamoebic Activity Assay

For all assays, harvested *E. histolytica* trophozoites in log phase were placed on 24-well plates (6 × 10^4^ trophozoites/well) in the presence of different concentrations of crude extracts (200 and 500 μg/ml) or fractions (18.75–300 μg/ml) and incubated at 37°C for 24, 48 and 72 h. Dimethyl sulfoxide (DMSO) was used as the solvent control, MTZ at 0.25 μg/ml was used as the amoebicidal drug control, and trophozoites with complete medium were used as the growth control. All wells had a final volume of 2.3 ml with complete medium. After incubation, trophozoites were detached by chilling in ice water for 30 min and counted on a hemocytometer. The viability was measured with trypan blue exclusion dye. The IC_50_ was calculated by probit analysis in IBM^®^ SPSS^®^ Statistics v. 25.

### Erythrophagocytosis Assay

*E. histolytica* trophozoites in the log phase of growth were harvested and incubated in 24-well plates (6 × 10^4^ trophozoites/well) with DMSO (0.1%), MTZ (0.25 μg/ml) or *C. sonorae* DMC fraction (118 μg/ml) every 24, 48 and 72 h. Trophozoites were washed twice with TYI-S-33 medium without supplementation and coincubated with human erythrocytes (1:100) every 30 min. Erythrophagocytosis was stopped with 4% paraformaldehyde to fix trophozoites. Phagocytized erythrocytes were counted in at least 80 amoebae per well. Trophozoites grown in complete medium were set as 100% erythrophagocytosis (Mora-Galindo et al., 2004).

### Transmission Electron Microscopy

To analyze ultrastructural changes, trophozoites treated with the *C. sonorae* fraction at 118 μg/ml (CsDCMfx) were harvested after 24, 48 and 72 h, washed twice with PBS (pH 7.2) and fixed with 2.5% glutaraldehyde in 0.1 M. Postfixation was performed with 2% osmium tetroxide. The samples were dehydrated with increasing concentrations of ethanol (70, 80, 90, 100%) and finally with propylene oxide. Subsequently, the amoebae were embedded in epoxy resin. Semithin sections (0.5 µm) were stained with toluidine blue and observed under a light microscope (Eclipse 80i microscope, Nikon, Japan). Fine sections (80 nm thick) were contrasted with uranyl acetate and lead nitrate and observed with a JEOL-JEM 1400 TEM (Pais-Morales et al., 2016).

### qRT-PCR Assays

Total RNA was extracted from *E. histolytica* trophozoites after 24, 48 or 72 h of incubation with DMSO (0.1%), MTZ (0.25 μg/ml), *Cs*DCMfx (118 μg/ml) or only complete medium (control) using an Arcturus PicoPure RNA Isolation Kit (Thermo Fisher Scientific, Lithuania) following the manufacturer´s instructions. After RNA quantification with a NanoDrop spectrophotometer, amplification of mRNA transcripts was performed using 100 ng of total RNA for the *calpain* gene and 0.1 ng for *actin*, *formin1*, *gal/galnac* lectin, *rho1*, *atg8*, *sod*, *enolase*, *pfor*, *cp1*, *cp2*, *cp4*, and *cp5.* qPCR with a SYBR Green RT-PCR one-step kit (Agilent, USA) was used. The primers used for amplification are shown in **Supplementary Figure 1** with *actin* as the reference gene. The PCR cycling conditions consisted of an initial step of 10 min at 50°C and 3 min at 95°C for all genes followed by 40 cycles of denaturalization at 95°C for 10 s and annealing at 60°C for 5 s for the *actin* (Majumder and Lohia, 2008), *formin1* (Majumder and Lohia, 2008), *gal/galnac* lectin (Ximénez et al., 2017), *rho1* (Bosch et al., 2011), *calpain* (Domínguez-Fernández et al., 2018), *sod* (Ximénez et al., 2017), *enolase* (Segovia-Gamboa et al., 2011) and *pfor* (Tazreiter et al., 2008) genes; 40 cycles of denaturalization at 95°C for 10 s, annealing at 52°C for 30 s and extension at 72°C for 30 s for *atg8* (Picazarri et al., 2015). The Ct values and the 2^−ΔCt^ formula were used for quantification (Livak and Schmittgen, 2001).

## Results

### Growth Curve of *E. histolytica*

It has been observed that pathogenic strains of *E. histolytica* trophozoites incur a fitness cost reflected in the doubling time *in vitro* (Calzada et al., 1998; Penuliar et al., 2015), which is longer than that of nonpathogenic strains and can be different between pathogenic strains. Due to these differences, it was necessary to construct a proliferation curve with different initial inocula (4, 6, 8 and 10 × 10^4^ trophozoites/well) counted every 24 until 96 h. The growth curve of *E. histolytica* showed that at 72 h, a maximum peak of growth was observed at 6, 8 and 10 × 10^4^ trophozoites/well, and the viability in all assays was greater than 95%, diminishing to almost half of the number of trophozoites at 96 h with viability under 90% (**Supplementary Figure 1**). The initial inoculum of 4 × 10^4^ trophozoites/well was discarded as an option to perform our experiments because it had a peak number of trophozoites at 96 h with viability under 90% (**Supplementary Figure 1**). Using the formula for doubling time in *E. histolytica* HM1:IMSS in this study, we obtained 26.05 ± 0.82 h until log phase (72 h) with 6 × 10^4^ trophozoites/well as the initial inoculum, and it was less variable between times for each initial inoculum, according to the growth curve (**Supplementary Figure 1**). Similar results were observed by Penuliar et al. (2015) using the same pathogenic strain (Penuliar et al., 2015). With these results, we selected 6 × 10^4^ trophozoites/well for all experimental procedures.

### Antiparasitic Activity of *C. sonorae* Extract

**Figure 1:**
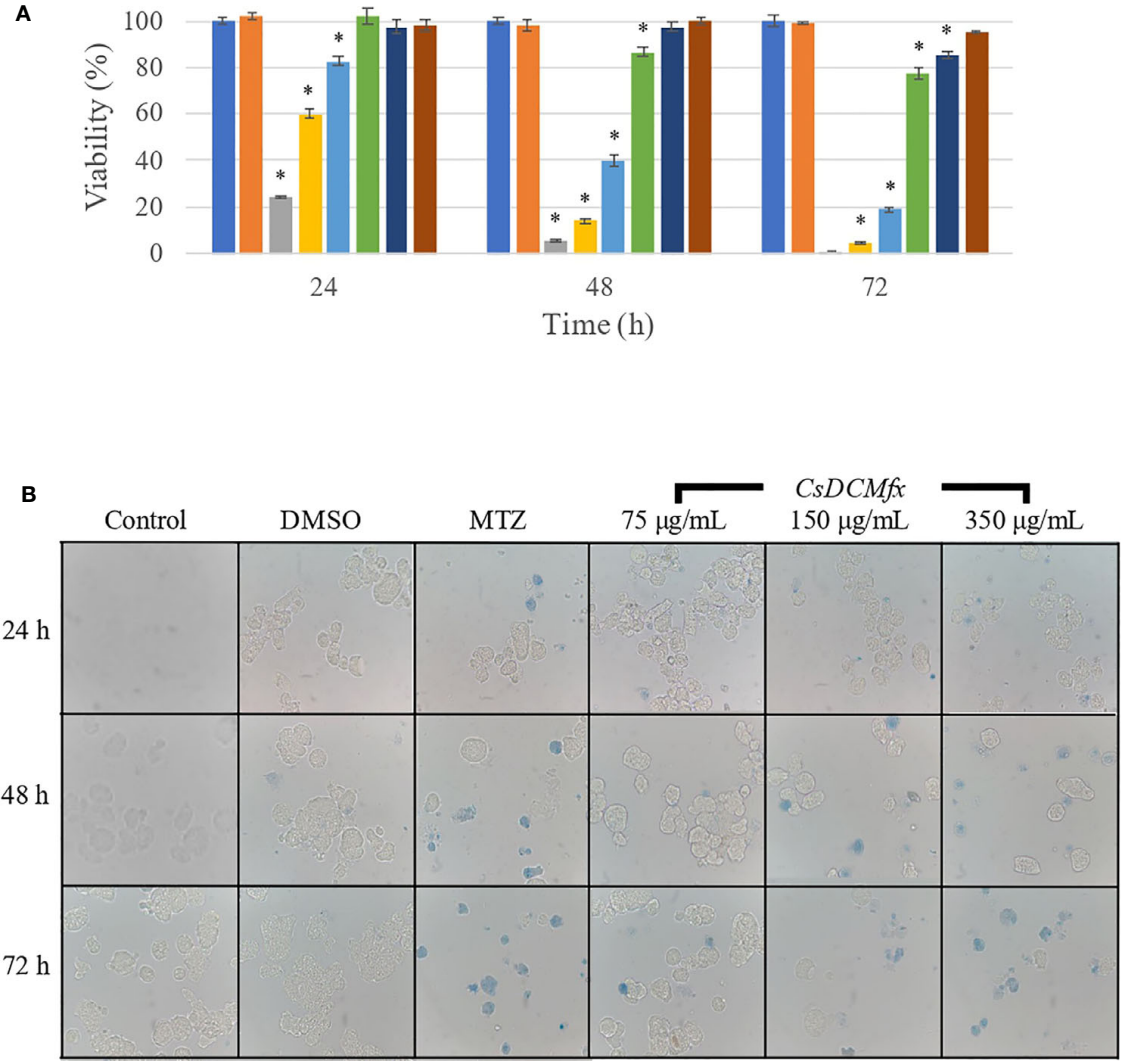
**(A)** Viability percentage of *E histolytica* trophozoites treated for 24, 48 and 72 h and cultivated with complete medium (control); MTZ was used as the drug control (0.25 μg/ml), DMSO as the diluent control (1%) and *C. sonorae* extract at different concentrations. **(B)** All samples were stained with trypan blue dye exclusion. 400×. Error bars represent SD. * means p <0.05 when compared with control. The blue bars represent control; orange bars represent DMSO; gray bars represent MTZ; yellow represent *C. sonorae* extract at 300 μg/ml; light blue represent *C. sonorae* extract at 150 μg/ml; green represent *C. sonorae* extract at 75 μg/ml; dark blue represent *C. sonorae* extract at 37.5 μg/ml; brown represent *C. sonorae* extract at 18.75 μg/ml.

To investigate the amoebicidal activity, we first evaluated the mortality percentage at 200 μg/ml and 72 h for crude plant extract, and the mortality was less than 5% for the extract evaluated in comparison with the control; the diluent control (DMSO) produced only 2.85% death, and MTZ at 0.25 μg/ml was ≥95% (**Supplementary Figure 2A**). Next, we tested 500 μg/ml plant extract, and *C. sonorae* extract produced mortality greater than 95% (**Supplementary Figure 2A**). To calculate the IC_50_ by probit analysis, we tested the *C. sonorae* extract at 200, 350 and 500 μg/ml, resulting in an IC_50_ = 306.23 μg/ml at 72 h. At 24 h with the *C. sonorae* extract (200 μg/ml), the trophozoites were agglutinated (**Supplementary Figure 2B**), but at higher concentrations (350 and 500 μg/ml), we observed increased mortality (**Supplementary Figure 2A**); at 72 h with the *C. sonorae* extract (200 μg/ml), trophozoites were smaller and rounded compared with control amoebae (**Supplementary Figure 2B**). We fractionated the *C. sonorae* extract with four different solvents: hexane, methanol, ethyl acetate and DCM. We tested them initially at 306 μg/ml, and only the hexane and DCM fractions produced mortality rates greater than 80% at 72 h (86.2 and 97.6%, respectively). To obtain the IC_50_ values for the hexane and DCM fractions, we tested 300, 150, 75, 37.5 and 18.75 μg/ml (**Figure 1A**). The calculated IC_50_ values were 118.7 and 122.1 μg/ml for the DCM and hexane fractions, respectively. The agglutination effect of the extract was lost in the hexane and DCM fractions at each time point and at all tested concentrations, but the trophozoites treated with CsDCMfx were affected in terms of shape and size similar to the extract (**Figure 1B**).

### Ultrastructure Changes of the Amoebae

**Figure 2:**
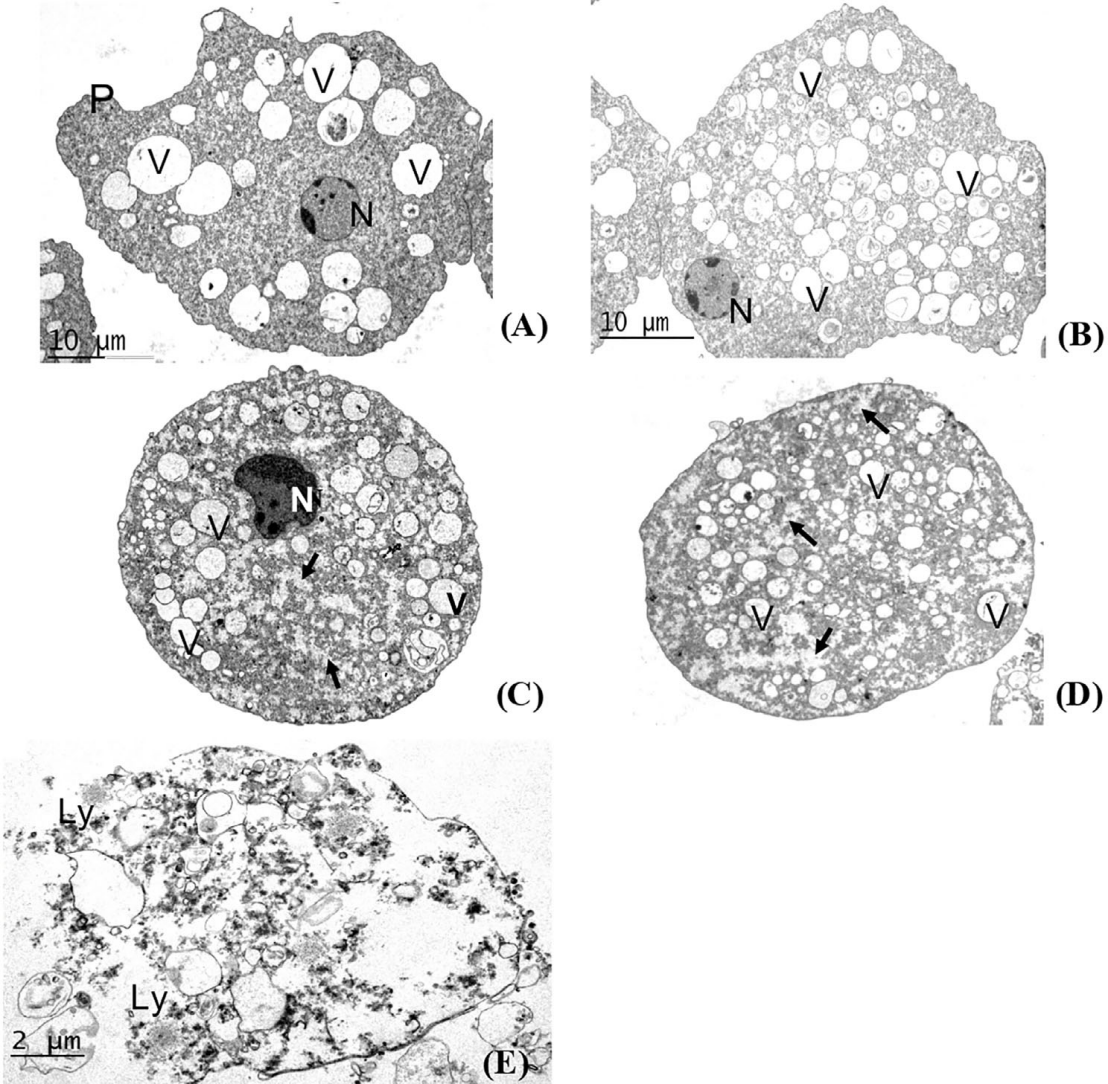
Transmission electron microscopy. **(A)** Control amoebae, without treatment. The amoeba presents typical morphology; a normal nucleus (N), pseudopod (P) and vacuoles (V) were seen. Bar = 10 µm.**(B)** Trophozoites with DMSO. The amoeba presents several vacuoles (V) of different sizes, the nucleus appears normal (N). Bar = 10 µm. **(C)** Trophozoite treated for 24 h with CsDCMfx. The trophozoite is rounded, presents abundant deposits of glycogen (arrows) and several vacuoles (V), and the nucleus shows abnormalities (N). Bar = 5 µm. **(D)** Amoebae treated for 48 h with CsDCMfx. The trophozoites present similar characteristics to those at 24 h of treatment. Bar = 5 µm. **(E)** Amoebae treated for 72 h with CsDCMfx. The ultrastructural morphology is completely abnormal, and lysis of the amoebae is evident (Ly). Bar = 2 µm.

The ultrastructure of the amoebae was analyzed by TEM. We found abundant glycogen deposits following treatment for 24 and 48 h with CsDCMfx (**Figures 2C, D**), and morphologically the cells appeared rounded and smaller than the control (axenic amoebae) and DMSO-treated cells (**Figures 2A, B**, respectively). The ultrastructural morphology of the amoebae treated for 72 h with CsDCMfx was completely abnormal, and lysis of the amoebae was evident (**Figure 2E**). The nuclei showed alteration of chromatin condensation, indicating a possible programmed cell death (PCD) in amoebae treated for 24 h. Resveratrol induced morphological alterations in the nucleus, vacuoles and cytoplasm similar to those changes caused by CsDCMfx (Pais-Morales et al., 2016).

### Erythrophagocytosis

**Figure 3:**
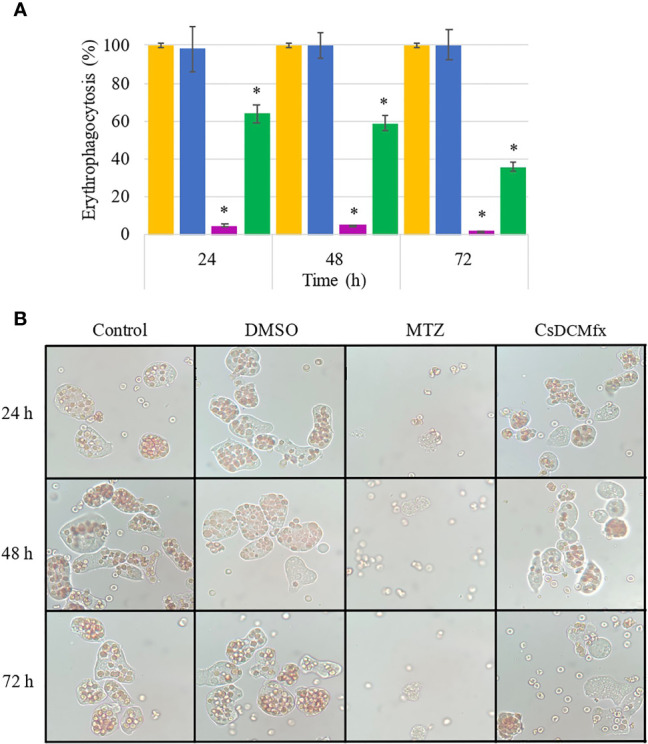
Erythrophagocytosis assay with average number of phagocytosed erythrocytes per group. **(A)** Control of amoebae was taken as 100% erythrophagocytosis for each time point to compare with DMSO (0.1%), MTZ (0.25 μg/ml) and CsDCMfx (118 μg/ml). **(B)** Representative images of each group of amoebae are shown. Error bars represent SD. * means *p* <0.005 when compared with the control. The orange bars represent control; blue bars represent DMSO (0.1%); pink bars represent MTZ (0.25 μg/ml); green bars represent CsDCMfx (118 μg/ml).

The actin cytoskeleton provides shape and motility in *E. histolytica* trophozoites; actin is recruited in the zone of contact with other cells to form the phagocytic cup and phagocyte (Majumder and Lohia, 2008; Herrera-Martínez et al., 2016). Because the *C. sonorae* DCM fraction (*Cs*DCMfx) affects the amoeba shape, we evaluated the phagocytic capability of trophozoites treated with CsDCMfx at 118 μg/ml for 24, 48 and 72 h. The phagocytic activity diminished significantly compared with control amoebae to 63.75% (SD ± 4.55%) at 24 h of treatment, then to 58.9% (SD ± 3.9%) and 35.73% (SD ± 2.26%) (48 and 72 h, respectively) (**Figure 3A**).

### mRNA Expression

**Figure 4:**
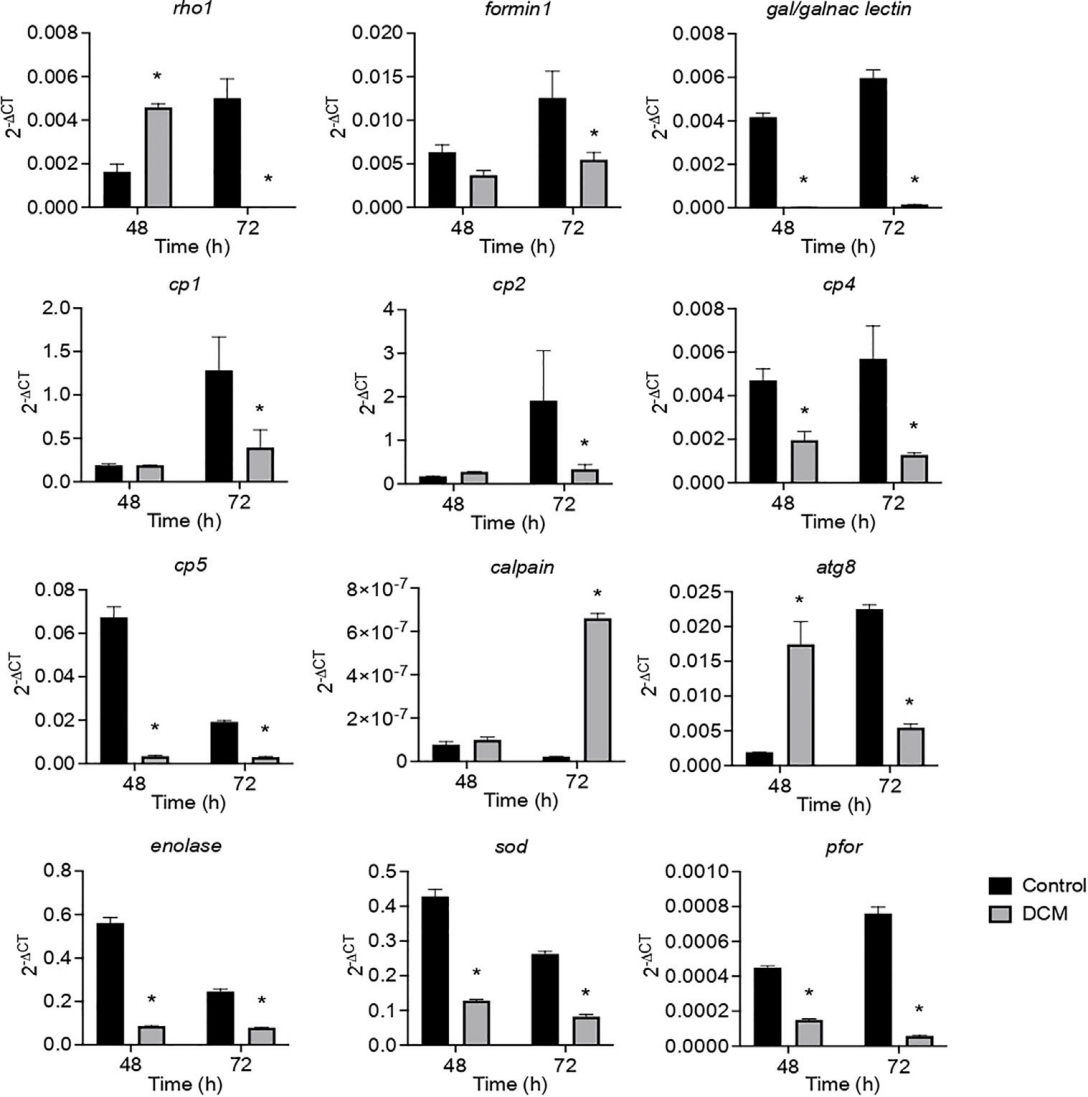
qRT-PCR of *rho1*, *formin1*, *gal/galnac lectin*, *cp1*, *cp2*, *cp4*, *cp5*, *calpain*, *atg8*, *enolase*, *sod*, and *pfor*. A 2^−ΔCT^ data analysis, taking C_T_ values for *actin* expression as the reference gene. DCM means amoebae treated with CsDCMfx at 118 μg/ml. Error bars represent SD. * means p < 0.05 when compared with control.

The amoebae treated with CsDCMfx for 48 h exhibited reduced mRNA expression of *formin1*, *gal/galnac lectin*, *superoxide dismutase* (*SOD*), *enolase*, and *pyruvate-ferredoxin oxidoreductase* (*pfor*) and increased expression of *Rho1*, *calpain* and *atg8* compared with control amoebae (**Figure 4**). The amoebae treated with CsDCMfx for 72 h exhibited increased expression of *calpain* in comparison with the other genes (**Figure 4**). We observed a decrease in *cps m*RNA in trophozoites treated with CsDCMfx.

## Discussion

To date, there has been no consensus regarding the cutoff point at which the concentration of an extract is considered to demonstrate good antiparasitic activity, as in the case of the antitumoral activity of extracts (<30 μg/ml) provided by the National Cancer Institute [33]. Here, we found that CsDCMfx is cytotoxic to *E. histolytica* trophozoites (IC_50_ = 118.7 μg/ml). This effect is considered “moderate” according to antiamoebic extract classification (Calzada et al., 2006); however, this particular classification (IC_50_ less than 20 µg/ml, the antiprotozoal activity was considered good, and from 20 to 150 µg/ml, the antiprotozoal activity was considered moderate) was defined by using a lower initial number of amoebae (6 × 10^3^) in comparison with the numbers used in this study (6 × 10^4^), which would underestimate the antiamoebic activity. We found the presence of some main secondary metabolite groups in the *C. sonorae* extract and CsDCMfx (Xu et al., 2018). Both the extract and CsDCMfx contain flavonoids previously identified and characterized in the genus *Croton* (Xu et al., 2018). It is known that some flavonoids can alter cytoskeletal functions in *E. histolytica* trophozoites and considerably diminish erythrophagocytosis activity (Bolaños et al., 2014; Bolaños et al., 2015). We observed in our results that erythrophagocytosis in amoebas treated with *Cs*DCMfx diminished (**Figures 3A, B**).

The proposed model for the role of Rho1 during phagocytosis in *E. histolytica* is that it recruits Formin1 by releasing it from an intramolecular inhibitory state. Formin1 dimerizes and forms F‐actin filaments beneath the membrane to generate a phagocytic cup (Majumder and Lohia, 2008). According to erythrophagocytosis assay and expression of *rho1* and *formin1*, the low expression of *formin1* (−1.73- and −2.30-fold change) can be a factor involved in the lack of phagocytosis in treated amoebae at 48 and 72 h (58.9 and 35.73%, respectively). The *gal/galnac* lectin was downregulated −105.1-fold at 48 h in amoebae treated with CsDCMfx, and this lectin is one of the principal adherence proteins facilitating the phagocytosis of red blood cells in phagocytic assays, supporting the lack of erythrophagocytosis. At 72 h, trophozoites treated with CsDCMfx and *gal/galnac* lectin expressed a −38.7-fold change, the *rho1* gene expressed a −175.6-fold change and *formin1* expressed a −2.30-fold change, explaining the lack of phagocytosis. It was demonstrated that in *E. histolytica*, upregulation of calpain-like genes occurs during programmed cell death (Domínguez-Fernández et al., 2018).

In this study, *calpain* was overexpressed 1.33- and 27.97-fold in amoebae treated with CsDCMfx at 48 and 72 h, respectively, suggesting an induction of programmed cell death, but according to the changes observed by TEM at 72 h, the amoebae died by lysis (**Figure 2E**). Autophagy is an intracellular degradation system that delivers cytoplasmic materials to the lysosome/vacuole (Nishimura and Tooze, 2020); *Entamoeba histolytica*, possesses a restricted set of autophagy-related (Atg) proteins compared with other eukaryotes; Atg8 is considered to be the central and authentic marker of autophagosomes (Picazarri et al., 2015). The *Atg8* gene was overexpressed in trophozoites treated with CsDCMfx at 48 h by 9.01-fold change, and it was downregulated by −6.44-fold change at 72 h.

In the *Croton* genus, more than 300 terpenoids have been identified and characterized (Xu et al., 2018). Velázquez-Domínguez et al. (2013) reported a sesquiterpene lactone, incomptine A, with an amoebicidal effect on *E. histolytica* and in energy metabolism, downregulating the protein expression of enolase and PFOR enzymes. We found that *pfor* and *enolase* mRNA was downregulated in the amoebae treated with CsDCMfx for 48 and 72 h.

SOD is the first line of defense against reactive oxygen species (ROS). SOD enzymes are a family of metalloenzymes responsible for quenching the potentially deleterious effects of superoxide radicals (Akbar et al., 2004). Elevated levels of superoxide radicals result in higher expression on iron-containing *sod* (Akbar et al., 2004). Our data showed downregulation of *enolase* and *sod* gene expression in amoebas treated with CsDCMfx at 48 h (−3.33- and −3.21-fold change, respectively). However, it is necessary to evaluate the presence of reactive oxygen species to confirm this evidence.

Cysteine proteases (CPs) play a key role in cleavage and penetration into the intestinal lumen. The genes of these proteins are overexpressed in *E. histolytica* but absent or nonexpressed in *E. dispar* (Ximénez et al., 2017). Through these proteins, CP1, CP2 and CP5 are responsible for 90% of the cysteine protease activity in *E. histolytica* (Siqueira-Neto et al., 2018). CP1 can digest collagen and adhere to enterocyte laminin, and it is located on the trophozoite surface and inside vacuoles (Nakada-Tsukui et al., 2012). CP2 is located beneath the internal membrane, and on the amoeba surface, it is capable of degrading collagen and cartilage. CP2 has been observed to be overexpressed *in vivo* using a hamster model for ALA development. The important implication of CP5 in virulence has been demonstrated: it is able to degrade mucin on colonic explants and is unable to invade the intestinal epithelium when *cp5* is silenced (Marie and Petri, 2014). CPs are necessary for the degradation of phagocytosed material, and we observed downregulation of *cps* in trophozoites treated with CsDCMfx (**Figure 4**) due to the lack of erythrophagocytosis. Further experiment will evaluate the proteinase activity to confirm the activity of CPs.

We also performed a transmission electron microscopy analysis to assess ultrastructural changes in *E. histolytica* induced by CsDCMfx (118 µg/ml) at different times. For control amoebae, without treatment, the amoebae presented their typical morphology, and a normal nucleus, pseudopods and vacuoles were observed (**Figure 2A**). *E. histolytica* trophozoites treated with 0.5% DMSO displayed normal morphology with several vacuoles and a nucleus containing peripheral chromatin (**Figure 2B**). After 24 and 48 h of treatment of *E. histolytica* with CsDCMfx, we observed several vacuoles of different morphologies and sizes, as well as increased glycogen stores. The continuity of the cytoplasmic membrane was preserved at these times (**Figures 2C, D**). After 72 h of treatment, disruption of the plasma membrane was evident, and glycogen and several vesicles were also observed (**Figure 2E**). Based on our transmission electron microscopy study, we believe that the mechanism of action of CsDCMfx against *E. histolytica* mainly affects the cytoplasmic membrane, causing lysis in the amoeba.

We consider it important to continue with the search and isolation of bioactive compounds of CsDCMfx that allow the generation of new pharmaceutical alternatives to treat amoebiasis. In conclusion, the plant *C. sonorae* is a potential source of antiamoebic compounds. CsDCMfx was able to prevent erythrophagocytosis by downregulating *gal/galnac* lectin, which is necessary for adhesion to erythrocytes, and actin rearrangement *via* the Rho1 GTPase and Formin1 pathways.

## Data Availability Statement

The original contributions presented in the study are included in the article/**Supplementary Material**. Further inquiries can be directed to the corresponding author.

## Author Contributions

OV: conception, project administration, supervision. IV-G and OV: writing original draft. MS and OV: visualization. IV-G, AS-O, RR-Z, JG-R, MS, and OV: investigation and data analysis. MS and OV: validation. IV-G and AS-O: methodology. RR-Z, JG-R, MS, and OV: resources. MS and OV: funding acquisition. All authors contributed to the article and approved the submitted version.

## Funding

This work was supported by the Consejo Nacional de Ciencia y Tecnología (CONACyT), Fondo Sectorial de Investigación para la Educación (grant number 258454). MS: CONACyT grant (grant number 237523).

## Conflict of Interest

The authors declare that the research was conducted in the absence of any commercial or financial relationships that could be construed as a potential conflict of interest.

## Publisher’s Note

All claims expressed in this article are solely those of the authors and do not necessarily represent those of their affiliated organizations, or those of the publisher, the editors and the reviewers. Any product that may be evaluated in this article, or claim that may be made by its manufacturer, is not guaranteed or endorsed by the publisher.

## Supplementary Material

Supplementary Figure 1
**(A)** Growth curve and viability **(B)** at 24, 48, 72 and 96 h with 4x10^4^, 6x10^4^, 8x10^4^ and 1x10^5^ trophozoites per well as initial inocula. Viability was measured with trypan blue exclusion dye. Error bars represent standard deviation (SD). The blue bars represent 4X10^4^ trophozoites/well; orange bars represent 6X10^4^ trophozoites/well; gray bars represent 8X10^4^ trophozoites/well; yellow bars represent 10X10^4^ trophozoites/well.

Supplementary Figure 2
**(A)** Viability percentage of *E. histolytica* trophozoites treated for 24, 48 and 72 h and cultivated with complete medium (control); MTZ was used as drug control (0.25 μg/ml), DMSO as diluent control (1%) and CsDCMfx at different concentrations. **(B)** All samples were stained with trypan blue dye exclusion. 400x. Error bars represent SD. *means p < 0.05 when compared with control.
