# Supplementary figures and images for: The Dichloromethane Fraction of Croton sonorae, A Plant Used in Sonoran Traditional Medicine, Affect Entamoeba histolytica Erythrophagocytosis and Gene Expression
Source: Front Cell Infect Microbiol. 2021 Jul 23;11:693449. doi: 10.3389/fcimb.2021.693449 (PMC8343225; doi:10.3389/fcimb.2021.693449)

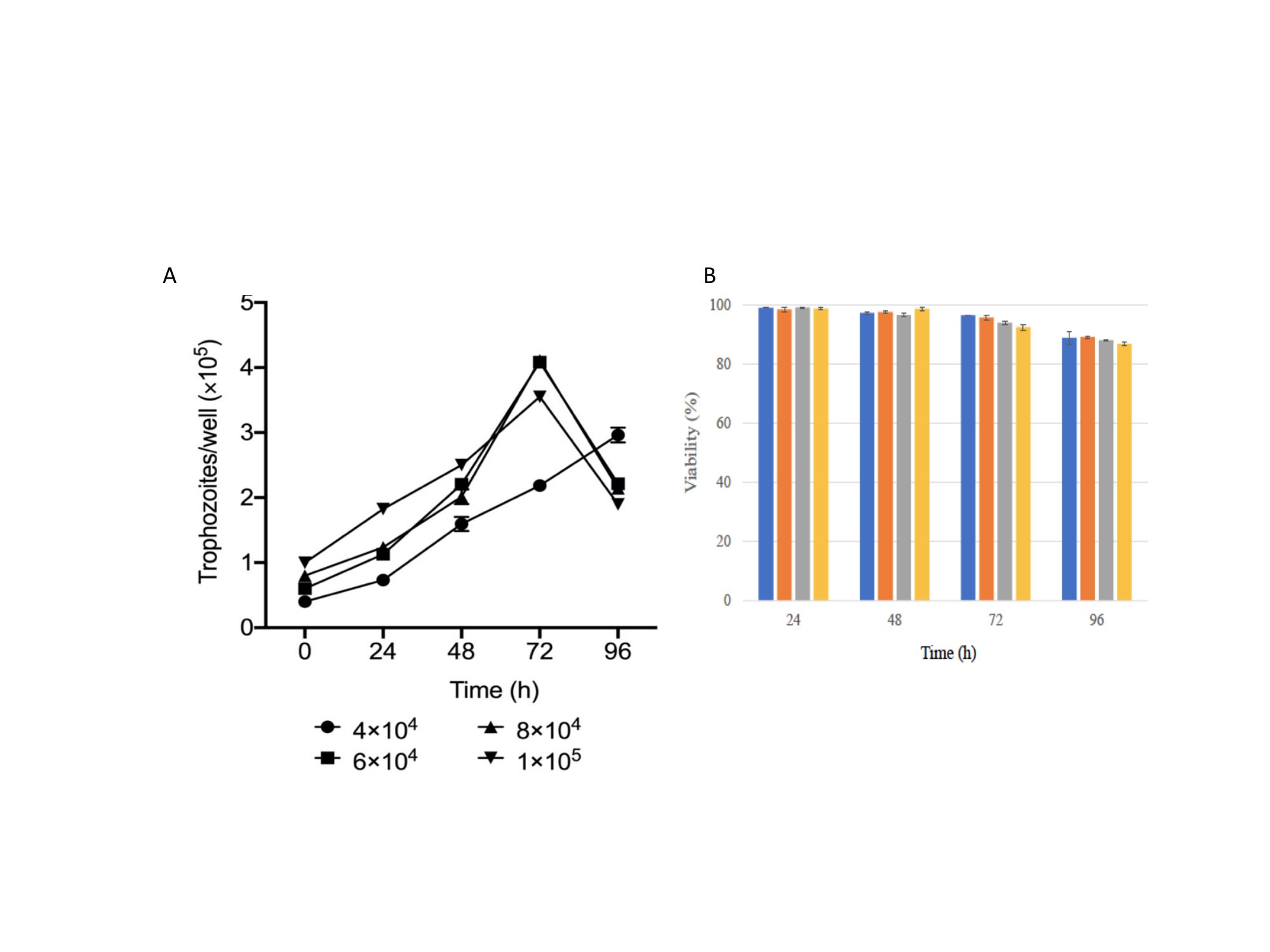

Supplement: Supplementary Figure 1 — (A) Growth curve and viability (B) at 24, 48, 72 and 96 h with 4x10^4^, 6x10^4^, 8x10^4^ and 1x10^5^ trophozoites per well as initial inocula. Viability was measured with trypan blue exclusion dye. Error bars represent standard deviation (SD). The blue bars represent 4X104 trophozoites/well; orange bars represent 6X104 trophozoites/well; gray bars represent 8X104 trophozoites/well; yellow bars represent 10X104 trophozoites/well. [file Image_1.jpeg]

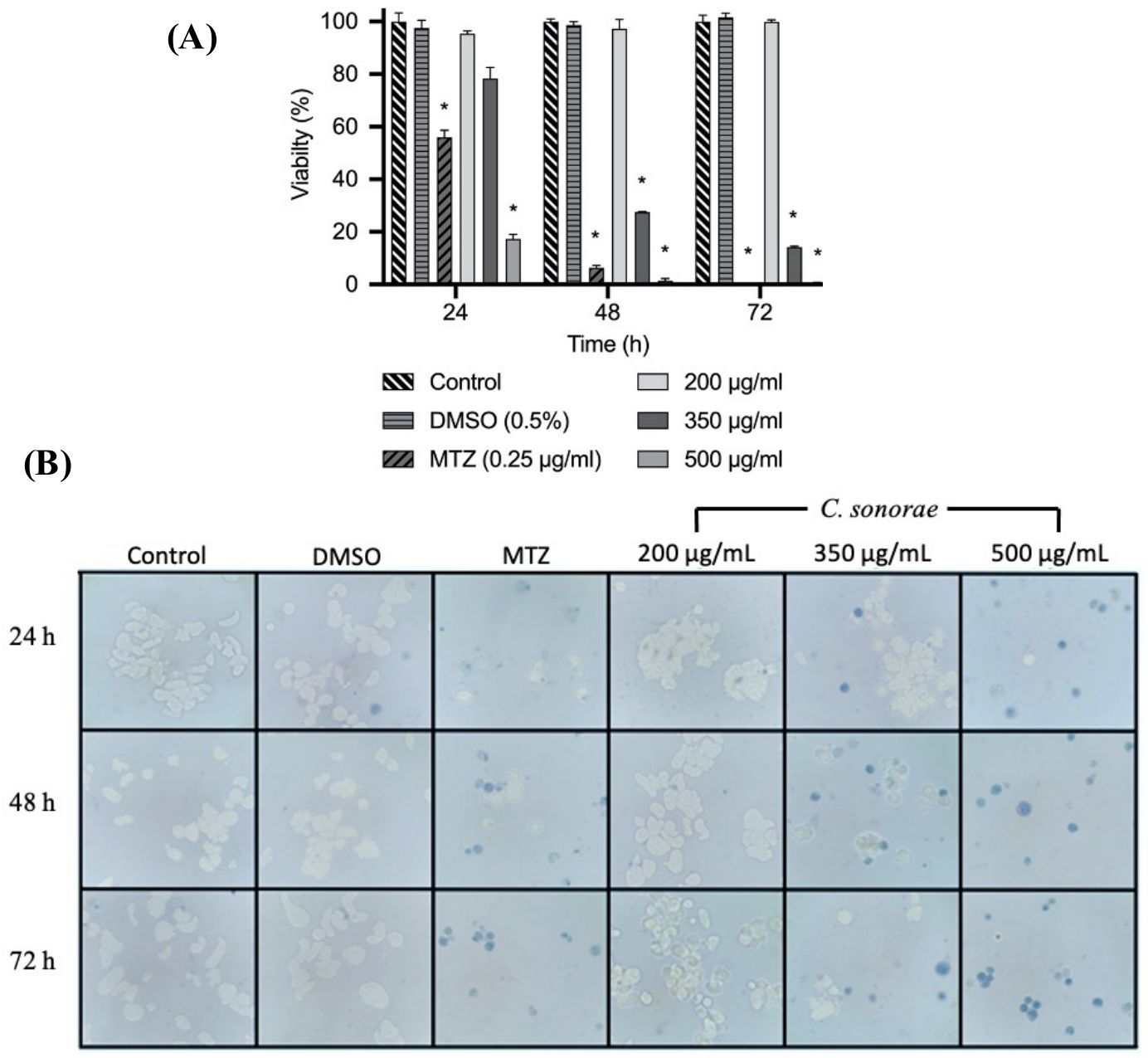

Supplement: Supplementary Figure 2 — (A) Viability percentage of E. histolytica trophozoites treated for 24, 48 and 72 h and cultivated with complete medium (control); MTZ was used as drug control (0.25 μg/ml), DMSO as diluent control (1%) and CsDCMfx at different concentrations. (B) All samples were stained with trypan blue dye exclusion. 400x. Error bars represent SD. *means p < 0.05 when compared with control. [file Image_2.jpeg]
